# Integrated Analysis of Multiple Microarrays Based on Raw Data Identified Novel Gene Signatures in Recurrent Implantation Failure

**DOI:** 10.3389/fendo.2022.785462

**Published:** 2022-02-07

**Authors:** Hong Zeng, Yu Fu, Lang Shen, Song Quan

**Affiliations:** ^1^ Department of Reproductive Medicine Center, Foshan Maternal and Child Health Care Hospital, Southern Medical University, Foshan, China; ^2^ Department of Gynecology and Obstetrics, NanFang Hospital, Southern Medical University, Guangzhou, China

**Keywords:** recurrent implantation failure, robust rank aggregation, microarray, differentially expressed genes, hub gene

## Abstract

**Background:**

Recurrent implantation failure (RIF) is an intricate complication following IVF-ET, which refers to the situation that good-quality embryos repeatedly fail to implant following two or more IVF cycles. Intrinsic molecular mechanisms underlying RIF have not yet been fully elucidated. With enormous improvement in high-throughput technologies, researchers screened biomarkers for RIF using microarray. However, the findings of published studies are inconsistent. An integrated study on the endometrial molecular determinants of implantation will help to improve pregnancy outcomes.

**Objective:**

To identify robust differentially expressed genes (DEGs) and hub genes in endometrium associated with RIF, and to investigate the diagnostic role of hub genes in RIF.

**Methods:**

Raw data from five GEO microarrays regarding RIF were analyzed. Integrated genetic expression analyses were performed using the Robust Rank Aggregation method to identify robust DEGs. Enrichment analysis and protein-protein interaction (PPI) analysis were further performed with the robust DEGs. Cytohubba was used to screen hub genes based on the PPI network. GSE111974 was used to validate the expression and diagnostic role of hub genes in RIF.

**Results:**

1532 Robust DEGs were identified by integrating four GEO datasets. Enrichment analysis showed that the robust DEGs were mainly enriched in processes associated with extracellular matrix remodeling, adhesion, coagulation, and immunity. A total of 18 hub genes (HMGCS1, SQLE, ESR1, LAMC1, HOXB4, PIP5K1B, GNG11, GPX3, PAX2, TF, ALDH6A1, IDH1, SALL1, EYA1, TAGLN, TPD52L1, ST6GALNAC1, NNMT) were identified. 10 of the 18 hub genes were significantly differentially expressed in RIF patients as validated by GSE111974. The 10 hub genes (SQLE, LAMC1, HOXB4, PIP5K1B, PAX2, ALDH6A1, SALL1, EYA1, TAGLN, ST6GALNAC1) were effective in predicting RIF with an accuracy rate of 85%, specificity rate of 100%, and sensitivity rate of 88.9%.

**Conclusions:**

Our integrated analysis identified novel robust DEGs and hub genes in RIF. The hub genes were effective in predicting RIF and will contribute to the understanding of comprehensive molecular mechanisms in RIF pathogenesis.

## Introduction

Implantation failure is the major limiting step in *in-vitro* fertilization and embryo transfer (IVF-ET) success. Approximately 15% of patients experience recurrent implantation failure (RIF) following IVF-ET. RIF refers to the situation that good-quality embryos repeatedly fail to implant following two or more ET cycles (1). Multifactorial pathogenesis plays a role in RIF, including embryo factors, endocrine factors, immunological problems, thrombotic conditions, uterine anomalies, genetic disorders, and endometrial factors (2). While the endometrial factor is one of the leading factors that account for RIF (3). However, intrinsic endometrial molecular mechanisms underlying RIF have not yet been fully elucidated. Endometrial gene expression profile may be disrupted in patients experiencing RIF (4). The value of identifying genetic biomarkers predictive of RIF is important as this would guide prognosis and inform potential therapeutic intervention for RIF. Increasing studies are investigating the endometrial gene expression profiles by microarray or RNA sequencing to identify biomarkers of prediction or diagnosis for RIF. Biomarkers including both coding genes and non-coding RNAs which are involved in implantation failure have been discovered and help to promote our understanding of pathogenesis underlying RIF (4–12). Nowadays, a tremendous amount of high-throughput data is piling up in public databases. However, results from the studies are inconsistent partly due to the small sample size in most studies or different platforms, or different bioinformatic methods. Most of these studies does not subject to validation on an independent cohort. Thus, there is an urgent need for timely identification and validation of more robust biomarkers for RIF, to improve pregnancy outcomes following IVF.

Biomarkers that are identified from a single study often appear to be biologically irrelevant or false positives. Meta-analysis allows integrating data from multiple studies to identify biomarkers across multiple conditions. Its main advantage is to boost power by increasing sample size and being able to catch signals that are small but consistent. Finding robust biomarkers for RIF is important for RIF prediction, diagnosis, and treatment. Researchers have characterized genetic cooperation and regulation through the menstrual cycle progression and characterized the genetic profiles for the acquisition of endometrial receptivity for a successful pregnancy (13). Recently, a panel of endometrial biomarkers acquired by endometrial receptivity test (ERT) was developed to accurately predict the window of implantation (WOI) and significantly improve the pregnancy outcomes of patients with RIF. These studies indicating the clinical potential of finding RIF related genetic profiles (14).

The purpose of the present study is to collect the available transcriptional microarray datasets from inconsistent measurements among various studies and perform an integrated analysis by robust rank aggregation (RRA) method (15) to generate more stable and robust DEGs. We perform protein-protein interaction analysis based on the robust DEGs to identify hub genes that may contribute to RIF, then validate the hub genes for prediction of RIF. Our study may help understand the mechanisms underlying RIF pathogenesis and promote the development of effective therapeutic targets of RIF.

## Materials and Methods

### Data Collection

**Figure 1:**
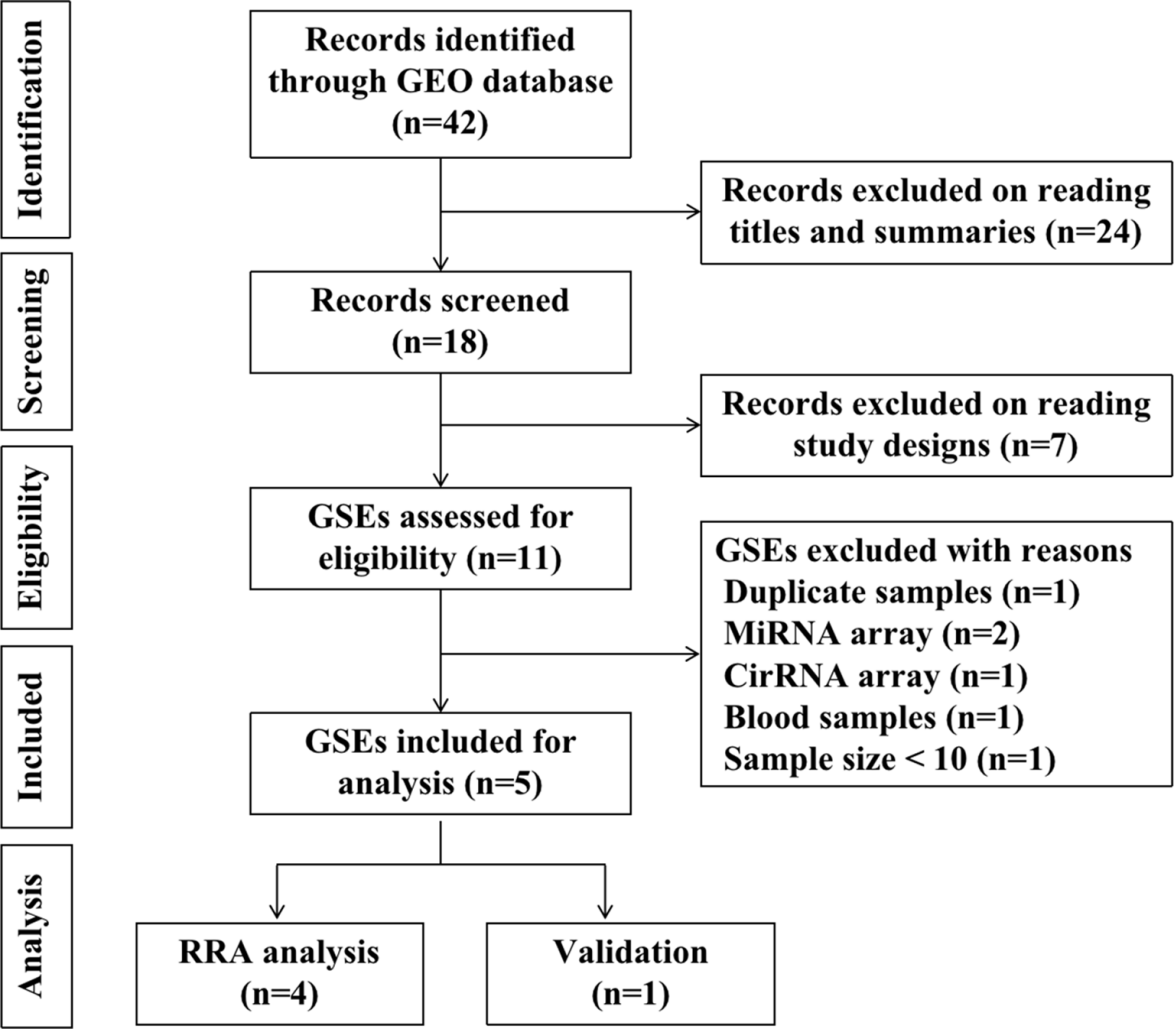
The diagram of selecting GSE datasets.

To identify gene expression data regarding recurrent implantation failure, we use the search term “implantation failure” to search gene expression datasets in Gene Expression Omnibus (GEO) database-series (https://www.ncbi.nlm.nih.gov/geo/browse/), The species are limited to humans. The searching keywords were “implantation homo”. Datasets that met the following inclusion criteria were included: (1) Gene expression profile by array; (2) The sample is the endometrium during the window of implantation; (3) Recurrent implantation patients and fertile controls were contained in one experiment; (4) The sample size is at least ten, with at least five patients in each group. (5) Raw data were available in GEO; (6) Chip platforms were from “Agilent” or “Affymetrix” or “Illumina”. By searching the GEO database, we identified 42 records in total. 24 records were excluded on reading titles and summaries, seven records were excluded on reading study designs. 11 GSEs were assessed for eligibility. Six GSEs were further excluded for the following reasons: duplicate samples and data (n=1, GSE71835); miRNA arrays (n=2, GSE71332, GSE108966); cirRNA array (n=1, GSE147442); blood samples (n=1, GSE106307); sample size < 10 (n=1, GSE103465). Finally, five GSEs (GSE26787, GSE92324, GSE111974, GSE58144, GSE71331) were included for statistical analysis. Among the five GSEs, four were included for RRA analysis, one was included in the validation and prediction process. The diagram of selected studies was shown in **Figure 1**. The following information were extracted from each identified GSE: GEO accession number, platform, sample size, array type, tissue, biopsy time, year, and country.

### Quality Control

The raw data were retrieved from the GEO database. Quality controls (QC) were conducted using the R-package ArrayQualityMetrics (16). Raw intensity signals (*.CEL files, *.txt files, *.idat files) were extracted and normalized using the R-package affy and vsn (17, 18). The datasets were annotated with the R Bioconductor packages hgu133plus2.db, hugene10sttranscriptcluster.db, and hgu133a.db, depending on the platform. We excluded non-annotated probes. The batch effect was removed by removeBatchEffect function in the limma package if the batch effect was significant (19). When multiple probes were mapped to the same gene, those probes were averaged. All codes run under the R environment 4.1.0

### Differentially Expressed Genes (DEG) Screening

The “limma” R package (19) was used to screen the DEGs between the RIF patients and the fertile control patients. The significantly DEGs in each GSEs were identified by p-value < 0.05 and |log2 fold change (FC)| >1. DEGs were ranked by p-value and |log2 FC|.

### RRA Analysis of DEGs From Different GSEs

The “RobustRankAggregation” R package (15) was used to integrate the ranked gene lists of DEGs to find the robust DEGs. The genes with adjusted p-value < 0.05 in the RRA analysis were considered as robust DEGs. For RRA analysis, we first analyzed the DEGs of each GSE dataset using the limma R package (19). Each DEG list was divided into the up-regulated gene list (log_2_FC > 0) and the down-regulated gene list (log_2_FC <0). DEGs (both up-regulated and down-regulated) of each dataset were ranked according to the p-value and |log_2_FC|. RRA for up-regulated genes and down-regulated genes was performed respectively. RRA is an effective tool to integrate multiple arrays outcomes. However, considering different array platforms, the input ranked gene lists only include the intersection of genes from the five GSEs. Then, all the DEGs were scored according to the ranked list and aggregately analyzed using the “Robust Rank Aggregation” R package. The final adjusted p-value in this method reflects the probability of the highly ranked genes in the datasets were identified as robust DEGs. We use the aggregateRanks() function to perform the RRA analysis. The parameters were set as the following: method=“RRA”, full=T, exact=T, topCutoff=NA. Details of the Robust Rank Aggregation (15) is as the following. For instance. let n be the number of experiments or GSEs and m be the number of genes studies. Assume that in DEG analysis of each GSEs genes are ordered according to their impact so that the genes locate in the beginning of the list are most likely to behave biologically functional. The rank of a gene is just the position in this ordering. If we divide ranks by the maximal rank value m, we obtained normalized ranks with the maximal value of 1. for each gene let the corresponding rank vector *r* = (*r*_1_,…, *r*_n_) be such that *r_j_* denotes the normalized rank of the gene in the *j*-th preference list. To find the genes that are highly ranked in m studied ranked lists. We assume that all that all informative normalized ranks come from a distribution, which is strongly skewed toward zero and our task is to detect these distributions. For any normalized rank vector *r*, let *r*_1_…, *r*_n_ be a reordering of *r* such that *r*_(1)_ ≤ … ≤ *r*_(_ *_n_*_)_ Then we can ask how probable it is to obtain ${\hat{r}}_{\left(k\right)}\leq \cdots \leq {\hat{r}}_{\left(k\right)}$ when the rank vector $\hat{r}$ is generated by the null model, i.e. all ranks ${\hat{r}}_{j}$ are sampled from uniform distribution. Let *β_k,n_* (*r*) denote the probability that ${\hat{r}}_{\left(k\right)}\leq {\hat{r}}_{\left(k\right)}$ . Then under the null model the probability that the order statistic ${\hat{r}}_{\left(k\right)}$ is smaller or equal to *x* can be expressed as a binomial probability


(1) $$\beta_{k,n}(x):=\Sigma_{\ell =k}^{n}\left({}_{\ell }^{n}\right)x^{\ell }{\left(1-x\right)}^{n-\ell },$$

since at least *k* normalized rankings must be in the range [0,*x*]. Alternatively, *β_k,n_* (*x*) can be expressed through a beta distribution, as ${\hat{\text{r}}}_{\left(k\right)}$ is the order statistic of *n* independent random variables uniformly distributed over the range [0,1]. Since the number of informative ranks is not known, we define the final score for the rank vector *r* as the minimum of *P*-values.


(2) $$\rho (r)=\underset{k=1,\ldots ,n}{\min}\beta_{k,n}(r)$$

The *ρ* score is a minimum of *β_n,k_* (*r*) scores, each of which is a p-value measuring deviance of the *k*-th order statistic ${\hat{r}}_{\left(k\right)}$ from its expected distribution. If the null hypothesis holds, then all the *β_n,k_* (*r*) values follow uniform distribution. If these values would be independent then the distribution of *p* scores would be *Beta*(1,*n*). We calculate the exact p-values based on*p* scores distribution. We want to calculate probability in the form


(3) Pr [X≤ρ]

Using the definition of rho we can write


$$\begin{array}{c} Pr\;[X\leq \rho ]=1-Pr\;[X\geq \rho ]\\ =1-Pr[\beta_{n,1}(\hat{r})\geq \rho ,\ldots ,\beta_{n,n}(\hat{r})\geq \rho ]\\ =1-Pr\;[{\hat{r}}_{\left(1\right)}\geq \beta_{n,1}^{-1}(\rho ),\ldots ,{\hat{r}}_{\left(n\right)}\geq \beta_{n,n}^{-1}(\rho )]\\ =1-Pr\;[1-{\hat{r}}_{\left(1\right)}\leq 1-\beta_{n,1}^{-1}(\rho ),\ldots ,1-{\hat{r}}_{\left(n\right)}\leq 1-\beta_{n,n}^{-1}(\rho )], \end{array}$$

where $\hat{r}$ is sample from uniform distribution with size *n* and $\beta_{n,k}^{-1}(\rho )$ is a quantile of *Beta*(k, *n – k* + 1) distribution. Therefore, we can write


(4) $$Pr\;[X\leq \rho ]=1-Pr[{\hat{r}}_{\left(1\right)}\leq 1-\beta_{n,n}^{-1}(\rho ),\ldots ,{\hat{r}}_{\left(n\right)}\leq 1-\beta_{n,1}^{-1}(\rho )]$$

The probability in (4) is the exact p-value.

### Gene Ontology (GO) and KEGG Pathway Enrichment Analysis

Gene Ontology (GO) and Kyoto Encyclopedia of Genes and Genomes (KEGG) pathway enrichment analysis were performed by the “clusterProfiler” R package (20). GO terms or KEGG pathways were visualized by the “ggplot2” R packages.

### Protein-Protein Interaction (PPI) Network Analysis

The RRA identified robust DEGs (exact p-value < 0.01) was employed to construct the PPI network. We uploaded the robust DEG list into the STRING database (http://www.string-db.org/), the confidence interaction score was set as 0.7 in the PPI analysis. The network was imported into the Cytoscape (version 3.8.2) (21) for visualization and further identification of hub genes. Hub genes are usually of functional importance and highly interconnected with other genes. We use the Cytohubba plug-in (22) to screen candidate hub genes. The scores of the genes were calculated by MMC, DMNC, EPC, and Degree methods. The genes were then ranked by scores according to MCC, DMNC, EPC, and Degree, respectively. The overlap of the top 100 ranked genes identified by Cytohubba following four methods and the top 100 robust DEGs were determined as the hub genes.

### Hub Genes Validation

We further conducted validation of hub genes by using the data from an Illumina microarray dataset (GSE111974) which consists of 24 RF patients and 24 fertile controls (8). The raw data were analyzed using the limma package (19). The background was corrected and the data was normalized. P-value <0.05 were considered to be significantly different.

### The Diagnostic Role of Hub Genes in RIF

We use the dataset GSE111974 (8) to investigate the diagnostic role of hub genes in RIF. The dataset was divided into the training set (60%) and the validation set (40%) by randomization with the seed number set as 1234. To verify the diagnostic role of hub genes identified by RRA and PPI analysis, we construct a prediction model using the differentially expressed hub genes by the generalized multivariate regression with the training set. First, we supply the training set into algorithm to construct the model. Second, we predict the markers of our validation set. Third, we calculate the number of correct and incorrect predictions on the validation dataset to assess the model’s prediction precision and calculate the accuracy rate, sensitivity rate, and specificity rate. Fourth. We performed the Receiver Operating Characteristic (ROC) analysis to detect the Area Under the Curve (AUC).

### Statistical Analysis

Continuous variables are presented as mean ± standard deviation (SD) for normally distributed data, or as median and interquartile range. Normally distributed data were compared using the Student’s t-test and non-normally distributed data using the Mann-Whitney U test. A p-value < 0.05 was considered to be statistically significant. All analyses were performed using R software (version 4.1.0). The R scripts and the corresponding Rdata file were deposited in our Github repositories (https://github.com/minizenghong/Genetic-meta-analysis-on-RIF).

## Results

### Characteristics of the Included GSE Datasets From the GEO Database

A total of five microarray datasets (GSE26787, GSE92324, GSE111974, GSE58144, GSE71331) met the inclusion criteria were included for subsequent analysis. There are 91 RIF patients and 114 control patients in the five datasets. The characteristics of the datasets included in this study were listed in **Table 1**. GSE26787, GSE92324, GSE58144, and GSE71331 were included in the RRA analysis. GSE111974 was selected for subsequent validation of hub genes and to investigate the diagnostic role of hub genes in RIF. The diagram of the study was shown in **Figure 1**.

**Table 1:** Characteristics of the included microarray datasets.

GSE ID	Platform	Participants (Control/RIF)	Array type	Tissue	Biopsy time	Year	Country
GSE26787	GPL570	5/5	Affymetrix	Endometrium	WOI	2011	France
GSE92324	GPL10558	8/12	Illumina	Endometrium	WOI	2016	India
GSE111974	GPL17077	24/24	Agilent	Endometrium	WOI	2018	Turkey
GSE58144	GPL15789	72/43	Agilent	Endometrium	WOI	2014	Netherlands
GSE71331	GPL19072	5/7	Agilent	Endometrium	WOI	2018	China

RIF, recurrent implantation failure; WOI, window of implantation.

### Identification of DEGs of Each GSE

**Figure 2:**
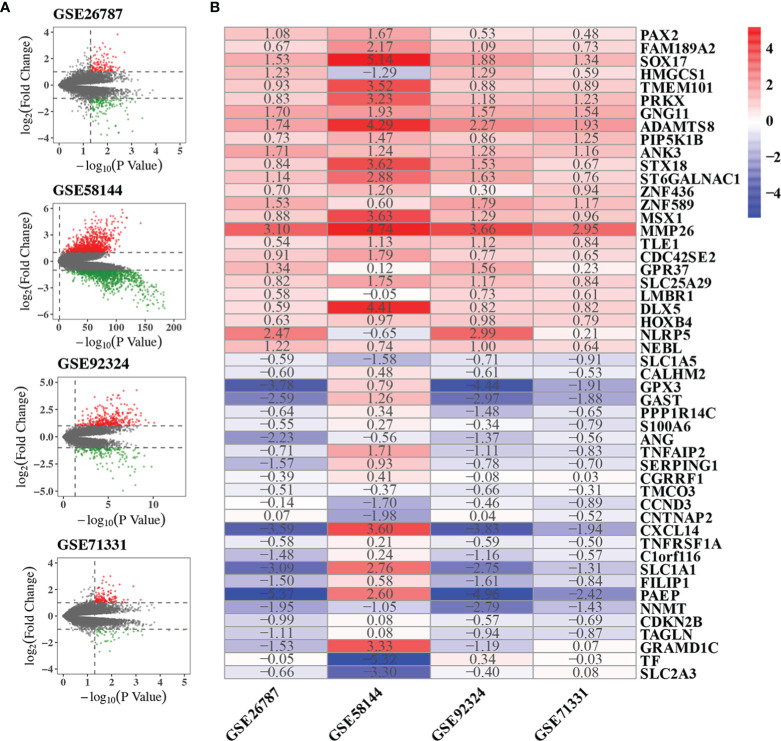
Results of RRA analysis. **(A)** Volcano plots showing DEGs of the four microarrays. Red points represented significantly up-regulated genes, while green points represented significantly down-regulated genes, grey points represented genes without significant difference. The criteria of DEG is |logFC|>1 and p-value<0.05. **(B)** Heatmap showing the top 25 up-regulated and 25 down-regulated robust DEGs in the RRA analysis. The number in box represents the log2FC. Red color denotes up-regulation, blue color denotes down-regulation, the color from dark to light represents the fold change from large to small.

We performed quality control, background correction, and normalization with the raw data from each GSE data set. Then DEGs of each GSE included in the RRA analysis were screened using the limma R package. The significantly DEGs were selected by p-value < 0.05 and |log2 FC| > 1. DEGs were ranked by p-value and |log2 FC|. The volcano plots showing DEGs of each GSE were shown in **Figure 2A**. The DEG list of each GSE dataset was shown in **Supplementary Table 1**. In summary, 121, 343, 1045 and 128 genes were significantly up-regulated in GSE26787, GSE92324, GSE58144, GSE71331, respectively. 156, 179, 1168 and 46 genes were significantly down-regulated in GSE26787, GSE92324, GSE58144, GSE71331, respectively.

### Integrative Analysis of DEGs From Different GSEs

GSE26787, GSE92324, GSE58144, GSE71331 were included in the RRA analysis. 1532 significantly robust DEGs were identified by the RRA analysis with the criteria of adjusted p-value<0.05. 438 significantly robust DEGs were identified by the RRA analysis with the criteria of adjusted p-value<0.01. The heatmap of the top 25 up-regulated and 25 down-regulated genes were shown in **Figure 2B**. The result of the RRA analysis was shown in **Supplementary Table 2**.

### Gene Ontology (GO) and KEGG Pathway Enrichment Analysis

**Figure 3:**
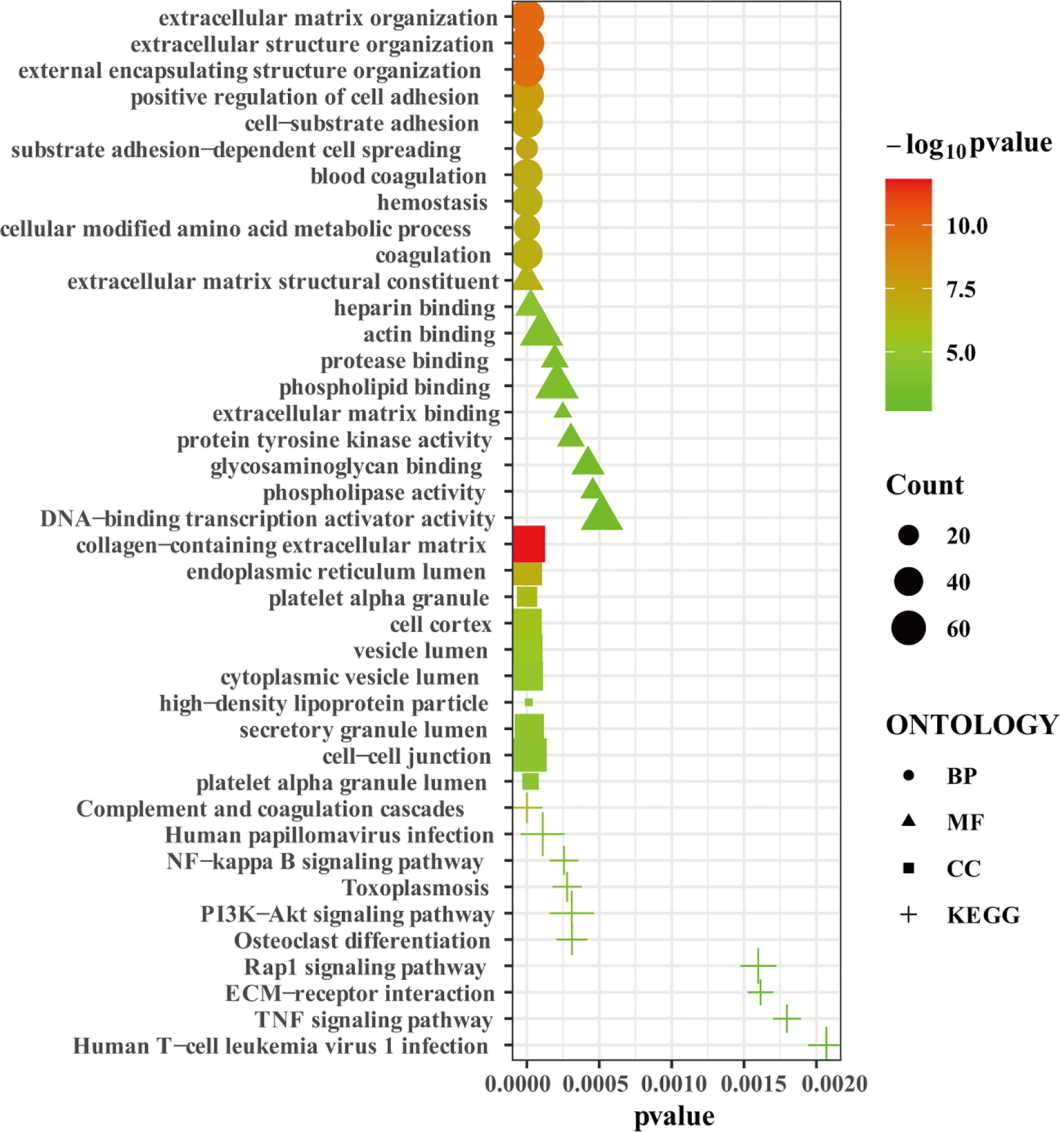
The GO and KEGG enrichment analysis. Bubble plot showing the top 10 enriched terms in biological process, molecular function, cell component, and KEGG pathways, respectively.

We uploaded the 1532 robust DEGs to perform the GO and KEGG enrichment analysis. GO enrichment analysis showed that extracellular matrix organization, extracellular structure organization, external encapsulating structure organization, positive regulation of cell adhesion, cell-substrate adhesion, substrate adhesion-dependent cell spreading, blood coagulation, hemostasis, cellular modified amino acid metabolic process, and coagulation were the top 10 enriched GO terms in biological process (BP) (**Figure 3**). Extracellular matrix structural constituent, heparin binding, actin binding, protease binding, phospholipid binding, extracellular matrix binding, protein tyrosine kinase activity, glycosaminoglycan binding, phospholipase activity, DNA-binding transcription activator activity were the top 10 enriched GO terms in molecular function (MF) (**Figure 3**). Collagen-containing extracellular matrix, endoplasmic reticulum lumen, platelet alpha granule, cell cortex, vesicle lumen, cytoplasmic vesicle lumen, high-density lipoprotein particle, secretory granule lumen, cell-cell junction, platelet alpha granule lumen were the top 10 enriched GO terms in cell component (CC) (**Figure 3**). KEGG enrichment analysis showed that complement and coagulation cascades, Human papillomavirus infection, NF-kappa B signaling pathway, Toxoplasmosis, PI3K-Akt signaling pathway, Osteoclast differentiation, Rap1 signaling pathway, ECM-receptor interaction, TNF signaling pathway, Human T-cell leukemia virus 1 infection were the top 10 enriched KEGG pathways (**Figure 3**). In summary, the robust DEGs were mainly enriched in processes associated with extracellular matrix remodeling, adhesion, coagulation, and immunity. The results of GO and KEGG pathway enrichment analysis was shown in the **Supplementary Table 3**.

### Protein-Protein Interaction (PPI) Analysis and Identification of Hub Genes

**Figure 4:**
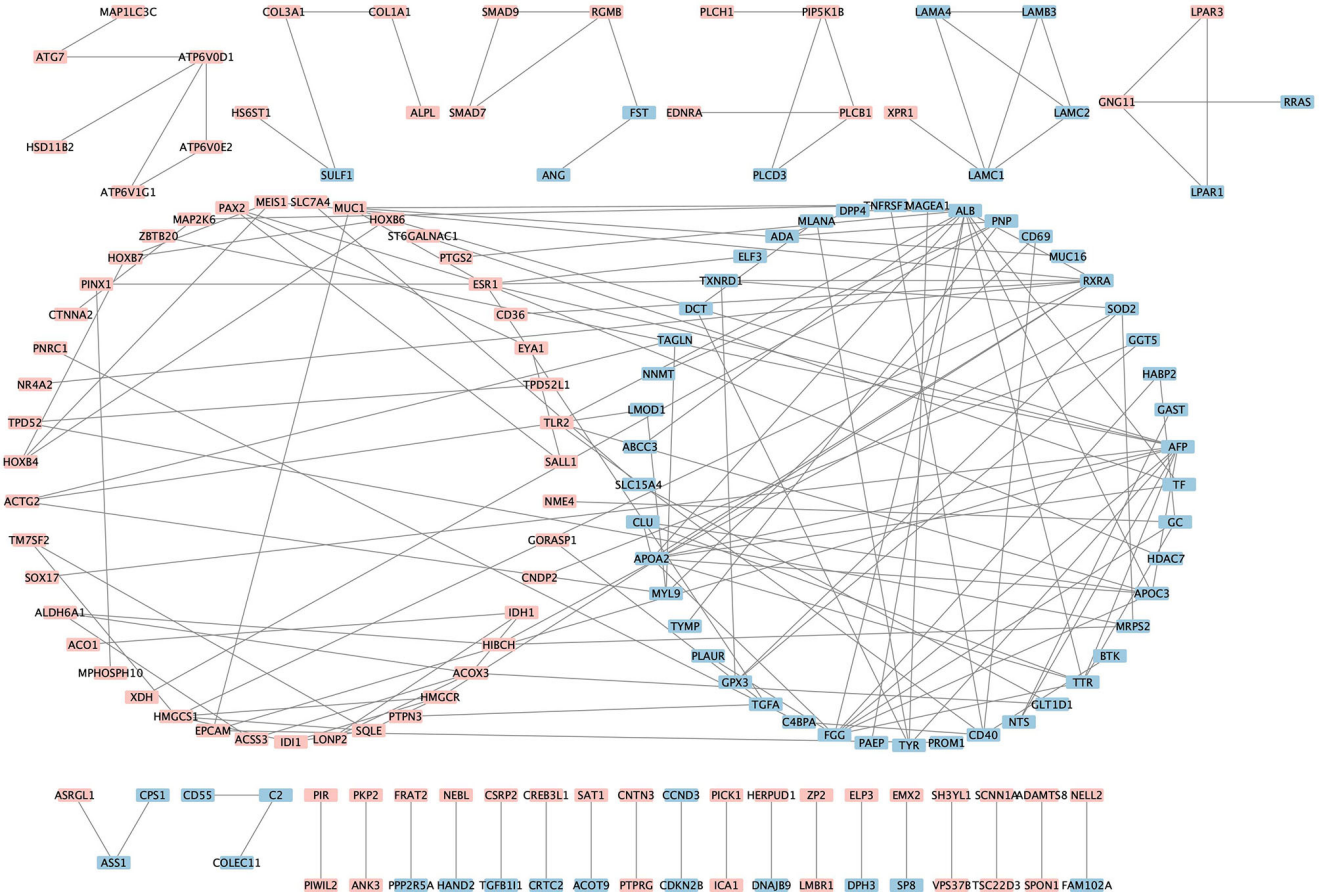
Visualization of the protein-protein interaction network. Pink denotes the up-regulated genes while blue denotes the down-regulated genes.

**Figure 5:**
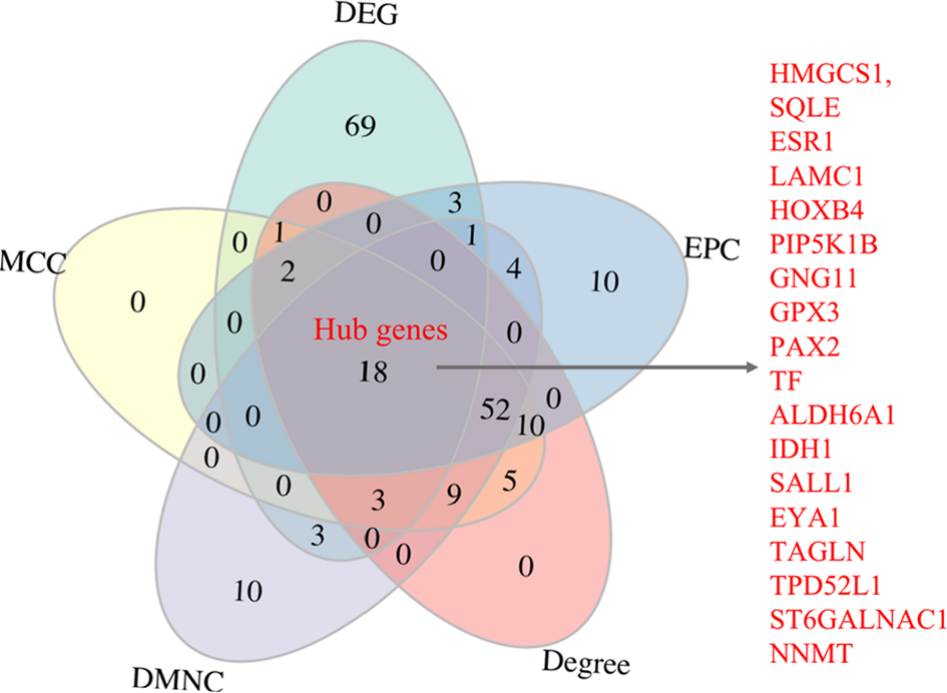
Venn plot showing the intersection of the hub genes.

The PPI analysis of the 438 robust DEGs identified from the RRA analysis (p-value < 0.01) was constructed using the String website and visualized by Cytoscape. The PPI network is comprised of 160 nodes and 174 edges (**Figure 4**). The interactions of the proteins were shown in **Supplementary Table 4**. The network was then imported into the Cytoscape for subsequent analysis. The CytoHubba plugin was used to identify the candidate hub genes in the PPI network. The nodes were ranked by scores following MCC, DMNC, EPC, and Degree methods, respectively. Results of the CytoHubba are listed in **Supplementary Table 5**. The top 100 ranked nodes were candidate hub genes. The overlap genes of the top 100 genes identified by Cytohubba following four methods and the top 100 robust DEGs in the RRA analysis were determined as the hub genes. Finally, 18 genes (HMGCS1, SQLE, ESR1, LAMC1, HOXB4, PIP5K1B, GNG11, GPX3, PAX2, TF, ALDH6A1, IDH1, SALL1, EYA1, TAGLN, TPD52L1, ST6GALNAC1, NNMT) were determined as the hub genes. The intersection of the top 100 genes identified by Cytohubba following four methods and the top 100 robust DEGs was shown by the Venn diagram in **Figure 5**.

### Hub Genes Validation

**Figure 6:**
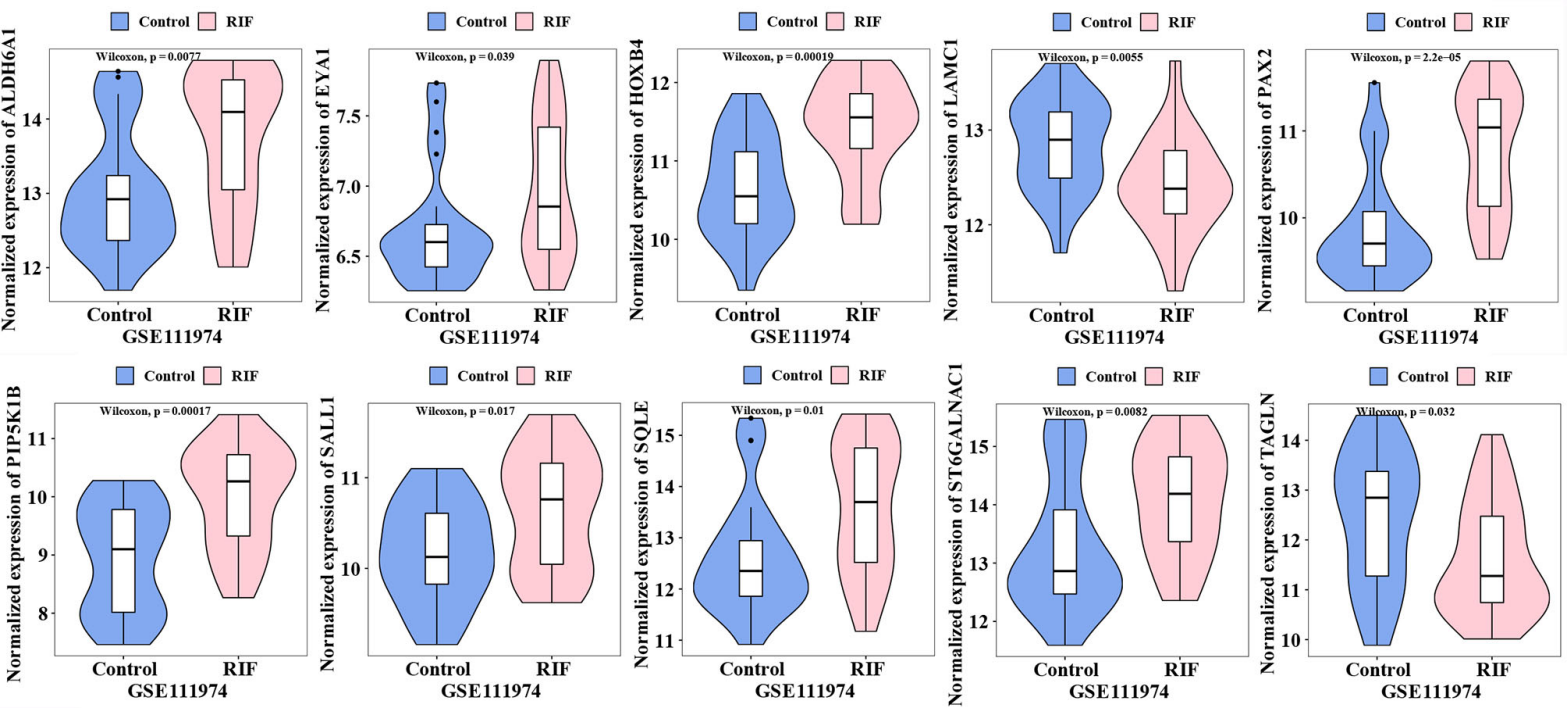
The normalized expression of hub genes validated in GSE111974.

The expression of the 18 hub genes was then validated in GSE111974. As the violin plots showed that ALDH6A1, EYA1, HOXB4, PAX2, PIP5K1B, SALL1 SQLE, ST6GALNAC1 were significantly increased in the RIF patients compared to control patients; while LAMC1, TAGLN were significantly decreased in the RIF patients compared to control patients (**Figure 6**). The other hub genes that were not significantly different between the RIF group and the control group were not shown in the violin plot.

### The Diagnostic Role of Hub Genes in RIF

**Figure 7:**
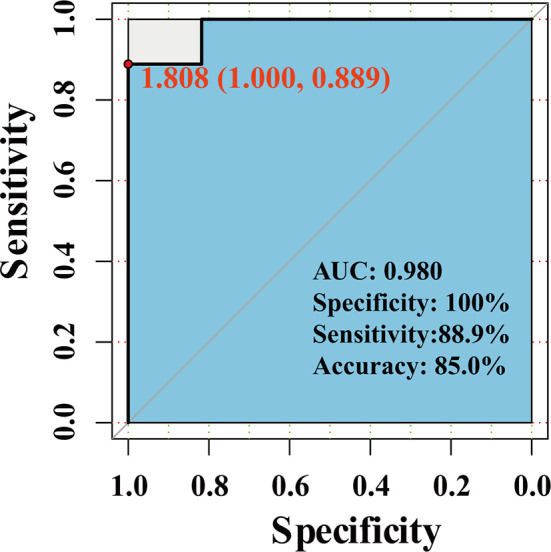
The Roc curve of the combined ten hub genes in predicting RIF.

We use the dataset GSE111974 to investigate the predictive effect of hub genes for RIF. 28 patients (13 controls and 15 RIF patients) were randomized divided into the training set, and 20 patients (11 controls and 9 RIF patients) were randomized divided into the validation set. In the validation set, the prediction model showed an accuracy rate of 85%, specificity rate of 100%, and sensitivity rate of 88.9%. The ROC analysis showed that the AUC is 0.980 (**Figure 7**).

## Discussion

Embryo implantation begins as the blastocyst hatching from the zona pellucida, followed by blastocyst apposition, adhesion, and invasion. Recurrent implantation failure (RIF) refers to the situation that good-quality embryos repeatedly fail to implant following two or more IVF cycles (1). The endometrial factor is one of the leading factors account for RIF (3). Therefore, identifying dysregulated genes in the endometrium of RIF is very important for understanding the pathogenesis of RIF and is clinically useful for RIF prediction. To the best of our knowledge, this is the first study to explore novel robust DEGs and hub genes in the endometrium associated with RIF by the RRA method combined with PPI and other bioinformatic tools. Robust DEGs were identified by integrating four array datasets. Consistent with published data, the enrichment of these robust DEGs in several terms such as extracellular matrix organization, adhesion, and coagulation were closely related to embryo implantation (23, 24). In addition, enrichment of these robust DEGs in some KEGG pathways, such as complement and coagulation cascades, Rap1 signaling pathway, ECM-receptor interaction, also suggest their relevance in implantation failure pathogenesis. From the enrichment analysis, we can conclude that the dysregulated genes in RIF were mainly involved in the processes associated with cell-cell and cell-matrix adhesion, ECM remodeling, coagulation, and immune response. All these processes are essential for embryo implantation (23, 24).

After the analysis of RRA, PPI, and Cytohubba, we eventually obtained 18 hub genes (including HMGCS1, SQLE, ESR1, LAMC1, HOXB4, PIP5K1B, GNG11, GPX3, PAX2, TF, ALDH6A1, IDH1, SALL1, EYA1, TAGLN, TPD52L1, ST6GALNAC1, NNMT). Some of them were demonstrated to play a role in regulating endometrial function and may be linked to embryo implantation. For example, LAMC1 is produced by the decidualized cells and serves as a key factor that controls decidual cell architecture and differentiation. Down-regulation of LAMC1 can prevent the formation of the basal matrix and lead to decreased trophoblast outgrowth (25). ESR1 can mediate estrogen effects and endometrial preparation for implantation, the expression or polymorphism of ESR1 was reported to be related to RIF (26). GPX3 plays a major role in reducing ROS during decidualization with its expression peaks during the implantation window. While inhibition of GPX3 is associated with a reduced pregnancy rate (27–29). TAGLN expression is significantly upregulated in the receptive endometrium compared to the pre-receptive endometrium (28) and is involved in regulating cell invasion, migration, and differentiation (30). One member of the TAGLN family, TAGLN2, is required for embryo implantation by promoting actin polymerization (31). NNMT in the uterine fluid is effective for the assessment of endometrial receptivity, NNMT protein in the uterine fluid was significantly decreased in RIF patients compared to fertile controls (32). IDH1 was expressed predominantly in the endometrial epithelia and involved in cellular defense against oxidative damage. IDH1 is required for a short time up to pregnancy recognition (33, 34). PAX2 is a known oncogenic gene in endometrial cancer, it is an estrogen-induced target gene (35). Abnormal increased PAX2 expression indicates over activation of the estrogen pathway which may impede embryo implantation. Consistently, our study confirmed that the expression of PAX2 was higher in RIF patients. Besides, some of the top25 up-regulated and down-regulated robust DEGs are reported to be associated with embryo implantation. ANG is an essential angiogenesis factor that plays important role in embryo implantation (36). SOX17 plays a key role in endometrial receptivity and embryo implantation by regulating embryo adhesion (37). MSX1 is critical for conferring uterine receptivity and readiness to implantation, it is significantly down-regulated in the receptive endometrium compared to the pre-receptive endometrium and reduced MSX1 in the endometrium is linked to infertility (28, 38–41). PAEP is also a marker for endometrial receptivity, PAEP was differentially expressed between RIF patients and fertile controls (7, 42). It is reported that receptive endometrial status can be determined based on the evaluation of mRNA expression levels of PAEP, DPP4, MSX1, and HLA-DOB genes (28). MMP-26 is localized in the epithelial cells of the human endometrium and its expression peaks in the early secretory phase. MMP-26 is reported to be an estrogen-sensitive gene with significantly higher expression at WOI in HRT cycles compare to natural cycles (43). Increased MMP-26 expression suggests overstimulation with E2. Consistently, our study indicates that the expression of MMP-26 was higher in RIF patients. SERPING1 mRNA is mainly in luminal and glandular epithelial cells and is significantly down-regulated in patients with recurrent miscarriage (44). SERPING1 is associated with decidualization and is involved in endometrial receptivity and immune regulation at the fetal-maternal interface (45–47). CXCL14 expression peaks at the embryo’s implantation site during WOI (48). CXCL14 is necessary to recruit natural killer cells (49) and is associated with a normal epithelial/stromal gene expression pattern (50). Cxcl14 knockout mice are infertile (51). It is reported that intrauterine hCG co-cultured with PBMCs administration induced expression of CXCL14 (52), while intrauterine injection hCG is reported to increase implantation rate of RIF patients (53). CCND3 expression increases upon decidualization progression and peaks at WOI (54), CCND3 is reported to regulate decidualization of uterine stromal cells during implantation (55, 56). SLC1A5 is likely responsible for increases in amounts of neutral and acidic amino acids in the uterine lumen to support conceptus growth, development, and survival (57).

In summary, by combining RRA, PPI, and Cytohubba analysis, we have successfully identified 18 hub genes associated with RIF and may provide deeper insight into the comprehensive molecular changes in RIF. Among the hub genes, ALDH6A1, EYA1, HOXB4, PAX2, PIP5K1B, SALL1 SQLE, ST6GALNAC1 were significantly increased in the RIF patients compared to control patients; while LAMC1, TAGLN were significantly decreased in the RIF patients compared to control patients in GSE111974 (**Figure 6**). A combination of the ten hub genes was effective in the prediction of RIF with an accuracy rate of 85%, specificity rate of 100%, sensitivity rate of 88.9%, and an AUC of 0.980 (**Figure 7**). Though novel robust DEGs and hub genes were identified, however, the underlying molecular mechanisms have not yet been fully elucidated. In the future, large cohorts are needed to validate the actual prediction power of the hub genes in the real-world population.

The strength of the study is that we obtained a larger dataset by combining data from four GEO datasets, which increased the sample size and ensured the stability and relative reliability of the conclusions. On the other hand, the RRA method was used to reduce the influences of the measurement platform, the sample size of datasets, the experimental design, and other factors on the final results. However, the potential limitations should also be underlined: (1) The validity of our conclusions mainly rests on the reliability of the original microarray datasets. (2) We applied GSE111974 to validate the expression of hub genes and to determine the diagnostic role of hub genes in RIF, however, the results were limited since the sample size is 48. More experiments are needed to validate the expression and function of hub genes in the future.

In conclusion, our integrated analysis identified novel robust DEGs and hub genes in RIF. The hub genes were effective in predicting RIF and will contribute to the understanding of comprehensive molecular mechanisms in RIF pathogenesis.

## Data Availability Statement

The datasets presented in this study can be found in online repositories. The names of the repository/repositories and accession number(s) can be found in the article/**Supplementary Material**.

## Ethics Statement

The study is an integrated analysis based on published datasets from Gene Expression Omnibus (GEO) database. Therefore, ethics approval is not applicable.

## Author Contributions

HZ designed the study, downloaded the raw data, performed the statistical analyses, draft the manuscript, tables and figures. LS and YF checked the tables and figures, revised the manuscript. SQ designed the study and revised the manuscript. All authors contributed to the article and approved the submitted version.

## Funding

This study is funded by the National Key R&D Program of China (grant number 2018YFC1004400).

## Conflict of Interest

The authors declare that the research was conducted in the absence of any commercial or financial relationships that could be construed as a potential conflict of interest.

## Publisher’s Note

All claims expressed in this article are solely those of the authors and do not necessarily represent those of their affiliated organizations, or those of the publisher, the editors and the reviewers. Any product that may be evaluated in this article, or claim that may be made by its manufacturer, is not guaranteed or endorsed by the publisher.

## Supplementary Material

Supplementary Table 1 DEGs in each microarray dataset.

Supplementary Table 2 Robust DEGs identified by the RRA analysis.

Supplementary Table 3 Results of GO and KEGG pathway enrichment analysis.

Supplementary Table 4 PPI interactions.

Supplementary Table 5 Results of the CytoHubba.
